# Surgical results of 158 petroclival meningiomas with special focus on standard craniotomies

**DOI:** 10.1007/s11060-022-04105-5

**Published:** 2022-09-14

**Authors:** Gabriele Schackert, Miriam Lenk, Matthias Kirsch, Silke Hennig, Dirk Daubner, Kay Engellandt, Steffen Appold, Dino Podlesek, Sahr Sandi-Gahun, Tareq A. Juratli

**Affiliations:** 1 Department of Neurosurgery, Carl Gustav Carus University Hospital, TU Dresden, Fetscherstr. 74, 01307 Dresden, Germany; 2 Institute of Diagnostic and Interventional Neuroradiology, Carl Gustav Carus University Hospital, TU Dresden, Dresden, Germany; 3 Department of Radiation Oncology, Carl Gustav Carus University Hospital, TU Dresden, Dresden, Germany

**Keywords:** Meningioma, Petroclival, Retrosigmoid and subtemporal, Pterional approach, Intraoperative tumor features, Classification, Complications

## Abstract

**Objective:**

The goal of this retrospective study is the evaluation of risk factors for postoperative neurological deficits after petroclival meningioma (PCM) surgery with special focus on standard craniotomies.

**Materials and methods:**

One-hundred-fifty-eight patients were included in the study, of which 133 patients suffered from primary and 25 from recurrent PCM. All patients were operated on and evaluated concerning age, tumor size, histology, pre- and postoperative cranial nerve (CN) deficits, morbidity, mortality, and surgical complications. Tumor-specific features—e.g., consistency, surface, arachnoid cleavage, and location—were set in a four-grade classification system that was used to evaluate the risk of CN deficits and tumor resectability.

**Results:**

After primary tumor resection, new CN deficits occurred in 27.3% of patients. Preoperative ataxia improved in 25%, whereas 10% developed new ataxia. Gross total resection (GTR) was achieved in 59.4%. The morbidity rate, including hemiparesis, shunt-dependence, postop-hemorrhage, and tracheostomy was 22.6% and the mortality rate was 2.3%. In recurrent PCM surgery, CN deficits occurred in 16%. GTR could be achieved in three cases. Minor complications occurred in 20%. By applying the proposed new classification system to patients operated via standard craniotomies, the best outcome was observed in type I tumor patients (soft tumor consistency, smooth surface, plane arachnoid cleavage, and unilateral localization) with GTR in 78.7% (p < 0.001) and 11.9% new CN deficits (p = 0.006).

**Conclusion:**

Standard craniotomies as the retrosigmoid or subtemporal/pterional approaches are often used for the resection of PCMs. Whether these approaches are sufficient for GTR—and avoidance of new neurological deficits—depends mainly on the localization and intrinsic tumor-specific features.

**Supplementary Information:**

The online version contains supplementary material available at 10.1007/s11060-022-04105-5.

## Introduction

Petroclival meningiomas (PCM) account for 2% of all meningiomas. PCM are located ventrally to the trigeminal nerve, have their origin on the dura mater of the petrous bone, and extend to the clivus, the petrosphenoidal ligament, the tentorium, or the sphenoid wing [1]. Because of the surrounding cranial nerves (CN) and the compression and adherence to the brain stem, the resection of these tumors is challenging and accompanied by a high risk of neurological deficits. Intensive discussions concerning appropriate skull base approaches were driven by the wish to expose the tumor and the surrounding CN as safely as possible, thereby avoiding complications. Regarding the origin and extension of the tumors, various tailored approaches have been favored [2–12]. However, complex skull base approaches are time-consuming in the opening phase and challenging during the closing procedure. Therefore, standard approaches, as well as their staged use, have been advocated [10, 13, 14].

The workhorse for tumors of the cerebello-pontine angle is the suboccipital retrosigmoid approach (RSA) [12–15]. This approach is easy to perform, done on a routine basis, and non-time-consuming. If the PCM is located purely infratentorially, the RSA can be sufficient for tumor removal. In cases, where the tumor is extending supratentorially, a two-stage procedure will be necessary by combining the suboccipital with a fronto-temporal craniotomy [10].Noteworthy, in PCM without significant lateral extension, endoscopic endonasal approach is considered by some neurosurgeons to achieve effective brainstem decompression [16].

The focus of this retrospective study is to evaluate the potential of standard craniotomies (the retrosigmoid and the subtemporal/pterional approach) for the resection of PCMs. Particular attention is paid to intrinsic tumor parameters that render these tumors resectable. Finally, a new classification system is used to assess the risk of CN deficits and the resectability of the lesions via standard approaches.

## Materials and methods

Between 1994 and 2021, 158 PCM patients were operated on in our department, 133 patients with the first manifestation and 25 patients with a recurrent tumor. The median observation period was 31 months (6–163 months).

### Indication for treatment

At first admission, all patients were informed about the standard treatment modalities, such as wait and scan, surgery, radiotherapy, or radiosurgery. If the patient was diagnosed for the first time and had only mild symptoms, we suggested an MRI control within half a year. Depending on the stability of the tumor size, neurological deficits, and patients´ wishes, we continued with MRI scans yearly. Surgery was indicated in large and growing tumors with compression of the brainstem, worsening of neurological symptoms, and patient´s desire. Primary radiotherapy or radiosurgery was suggested in cases of small, non brainstem compressing lesions or upon the patient´s wish. Since the establishment of our interdisciplinary tumor board in 2006, all patients were interdisciplinarily discussed and the recommendations were followed.

### Surgical procedure

The selection of the approach depended on the adhesion zone and the largest extension of the tumor with compression of functional cerebral structures. A preoperative embolization was not performed in any of the cases. In most cases, we started with the retrosigmoid approach to decompress the brainstem.

For the retrosigmoid approach, we positioned all patients in the lateral decubitus position. In cases where the tumor extended to the middle and anterior fossa, the remaining tumor was removed via a pterional or fronto-temporal approach in a second session**.** In these cases, the patient was transferred in a supine position. The interval between the first and second surgery was at least three months. In the rare cases, in which we used the combined pre-retrosigmoid or presigmoid-subtemporal approaches the patients were positioned in the lateral position.

Generally, the first step was to disconnect the tumor from its dural matrix for devascularization, and the second step was to reduce the tumor mass by using surgical ultrasound or by piecemeal removal. If an arachnoid cleavage was preserved and the tumor was soft, an adequate gross total tumor resection was possible, while leaving the vascular structures and nerves unharmed. If the surface of the tumor was like a cauliflower, major attention had to be paid to the small rami ad pontem, which could be hidden behind the uneven tumor surface. For the preparation of the vascular perforators or cranial nerves, we used a microdissector, a 135° angled little hook with a ball point on the top, or the fine preparation forceps, alternatively. In most cases, the cranial nerves and the vasculature could be stripped off in the arachnoid layer. If radical resection was too risky, we decided against complete tumor removal for the sake of maintaining neurological function and left a small tumor remnant. In firm tumors, the resection was more difficult. Even tumors with a plain surface were hard to handle. In many cases, a surgical ultrasound was not able to reduce the tumor mass. All pieces had to be cut by scissors or even scalpels. In some instances, it was impossible to reduce the tumor to a significant degree. Electrophysiology was applied on a routine basis for monitoring the cranial nerves 7, 8, and 9 as well as the somatosensory and motor evoked potentials (SSPE and MEP).

The preparation of recurrent tumors is more difficult with respect to scares that engulf cranial nerves and vessels. If no tumor was hidden behind these scars, we left them behind and concentrated on the removal of new tumor tissue, which was in many cases soft and could easily be removed or even sucked off by the ultrasound aspirator.

Since the facial, the vestibulocochlear, and trigeminal nerves are of special risk in the retrosigmoid approach because of their location on the dorsal surface of the tumor and therefore in the surgical path, we tried to avoid stretching these nerves by inserting the spatula on the upper surface of the cerebellum and then going to the lateral surface. In any case, tumor consistency was crucial for tumor removal.

### Adjuvant radiotherapy or radiosurgery

In case of incomplete resection, histology of atypical or anaplastic meningeoma or recurrent tumors postoperative irradiation was discussed. Conventional fractionated stereotactical radiotherapy with photon beam therapy or since 2015 with protons was given to a dose of 54–60 Gy in 27–30 fractions depending on the histology and the proximity to critical structures like brainstem or optical system. The treatment volume consists of the prior tumor volume with a safety margin of 1–2 cm along the meninges. Radiosurgery was applied by use of cyberknife, the dosis was in relation to the size of the tumor [17].

### Outcome parameters and follow-up

According to the EORTC and RTOG, Simpson grade I-III were defined as GTR, Simpson grade IV as subtotal resection, and Simpson grade V as biopsy.

All patients were evaluated with respect to pre- and postoperative CN deficits and hemiparesis. Postoperatively, the extent of tumor removal on MRI, major complications, and mortality were analysed. Due to the large observation period of the study, the time-point for evaluation of CN deficits varied. The minimum follow-up time was six months post surgery [18].

### Intraoperative tumor features proposed classification system

Based on the intraoperative tumor features, we grouped the tumors according to their location and specific tumor parameters, which are: extension of the adhesion zone, tumor texture, surface, and arachnoid cleavage.

Type I: dural adhesion zone restricted to one side of the posterior and middle fossae, soft tumor consistency, smooth surface, intact arachnoid layer (Supp. Figure 1).

Type II: dural adhesion zone restricted to one side of the posterior and middle fossae, intermediate to firm tumor consistency, smooth surface, intact arachnoid layer (Supp. Figure 2).

Type III: dural adhesion zone restricted to one side of the posterior and middle fossae, firm tumor consistency, cauliflower surface, w/wo arachnoid cleavage and brainstem edema (Supp. Figure 3).

Type IV: bilateral dural adhesion zone restricted mainly to the clivus, firm tumor consistency, smooth or cauliflower surface, w/wo arachnoid cleavage and brainstem edema (Supp. Figure 4). This type represents mainly clival meningiomas.

Patients operated via standard approaches (RSA and subtemporal/pterional) resembled the two largest equally treated groups. Both cohorts were additionally investigated with respect to CN deficits and GTR according to the proposed classification system.

### Correlation of intraoperative tumor features with preoperative MRI scans

Preoperative MR scans from 70 patients were assessed for T2-weighted (“hyper-intense”/“hypo-intense”) signals, using a previously reported approach [19, 20]. The signal in T2-weighted images (T2 relaxation time) was correlated with the intraoperative tumor consistency. In comparison with the intensity of the normal cortex, firm tumors were reported to have a lower intensity on T2-weighted images, whereas soft tumors are hyperintense [21]. The assessment was performed by two independent clinically experienced neuroradiologists (D.D. and K.E.). Both reviewers were blinded to the intraoperative tumor consistency.

### Statistical analysis

Statistical analyses were performed using the statistical software SPSS (27^th^ version, IBM Analytics). The data were analyzed using the Mann–Whitney U test and Fisher's exact test, according to the characteristics of the dataset. A 2-sided P-value < 0.05 was considered to be statistically significant. The diagnostic test evaluation calculator was used to calculate the sensitivity, specificity, positive predictive value, and negative predictive value (https://www.medcalc.org/calc/diagnostic_test.php (Version 20.106; accessed May 3, 2022).

## Results

### Surgical approaches

The RSA was used in 101 primary and 10 recurrent cases. Alternative craniotomies were the subtemporal/pterional (n = 38), the combined pre-retrosigmoid (n = 7) and combined presigmoid/subtemporal (n = 2). The supracerebellar transtentorial approach was not used. Two-stage surgery was necessary in 15 cases (Table 1). The decision for two-stage surgery was made a priori.

**Table 1:** Patients demographics and tumor features in the primary and recurrent tumor cohort

∑ 158 PCM	Primary surgery	Recurrence surgery
	(n= 133 patients)	(n= 25 in 20 patients)
Median age (Range)	60.9 (19.4–81.6) years	65.4 (22–85) years
Male/female [n (%)]	28 (21.1 %)/105 (78.9%)	5 (25%)/15 (75%)
WHO grades 1/2/3 [n (%)]	114 (85.7%)/ 15 (11.3%)/ 0 (0%)	14 (56%)/ 6 (24%)/ 2 (8%)
Histology [n (%)]		
Meningothelial	74 (55.6%)	10 (40%)
Fibrous	18 (13.5%)	1 (4%)
Transitional (mixed)	17 (12.7%)	3 (12%)
Angiomatous	2	
Psammomatous	1	
Microcystic	1	
Secretory	1	
Atypical	13 (9.7%)	6 (24%)
Clear cell	1	
Chordoid	1	
Anaplastic		2 (8%)
No data	4 (3%)	3 (12%)
Median tumor diameter (range)	3.2 (1.3–6.8) cm	4.0 (2.8–6.7) cm
Cavernous sinus infiltration [n (%)]	38 (28.6%)	17 (68%)
Two-stage surgery [n (%)]	13 (9.8%)	2 (8%)
Surgical approach [n (%)]		
Subtemporal/pterional	24 (18.0%)	14 (56.0%)
Suboccipital retrosigmoid	101 (75.9%)	10 (40.0%)
Combined pre-/retrosigmoid	7 (5.2%)	–
Combined presigmoid/subtemporal	1	1
Gross total resection	79 (59.4%)	3 (12%)

### Primary petroclival meningiomas (n = 133)

The median age of patients at primary diagnosis was 60.9 years (19.4–81.6 years). The gender distribution was 105 women and 28 men. The median tumor diameter was 3.2 cm (1.3–6.8 cm). Patients’ demographics and tumor features are shown in Table 1.

GTR was confirmed by postoperative MRI and was achieved in 59.4% (Table 1). Spanning three WHO classification periods (1993, 2007, 2016), one-hundred-fourteen meningiomas were graded as WHO grade 1. The meningothelial type was diagnosed in 74 patients, the fibrous type in 18, and the transitional type in 17 patients, representing the most common histological subtypes (Table 1). Fifteen meningiomas were classified as WHO grade 2 tumors, of which 13 were atypical, one was clear cell and one was chordoid.

#### (a) Preoperative symptoms

**Fig. 1:**
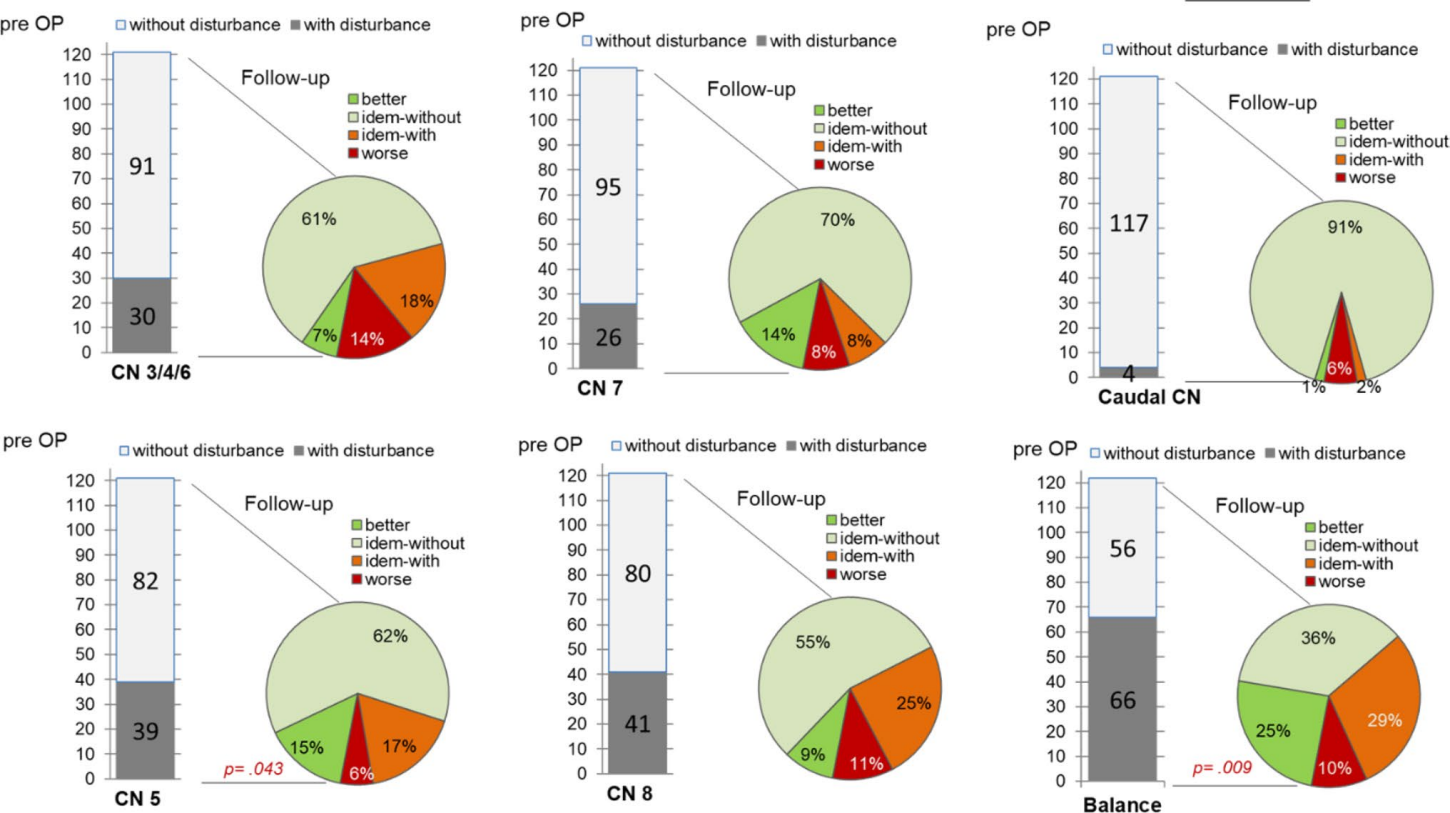
Preoperative neurologic deficits and at follow-up after primary PCM surgery demonstrated are individual symptoms of the CN 3–9 and of balance impairment. In all subfigures on the left depicted are the distributions of preoperative symptoms in a column bar, compared to the follow-up course in the right-handed pie chart punction

The leading preoperative symptoms were ataxia (n = 66) and CN deficits, of which symptomatic trigeminal neuralgia (n = 39) and hearing loss (n = 41) had occurred most frequently. Thirty patients complained about oculomotor nerve deficits CN3/4/6 (double vision, paresis of the levator palpebrae muscle, ophthalmoplegia). A CN7 paresis (HB II and III) had occurred in 26 patients and four patients reported dysphagia (Fig. 1).

#### (b) Postoperative outcome

Of 133 operated patients, 121 could be evaluated by a follow-up period of at least 6 months. Each of the neurological functions was separately evaluated. Patients’ ataxia improved in 30 (25%) and worsened in 12 patients (10%, p = 0.009). CN3/4/6 pareses occurred in 17 patients (14%), whereas 8 (7%) improved. CN5 deficits resolved in 18 patients (15%), and worsened in 7 (6%, p = 0.043). New CN7 paresis (HB III–V) occurred in 9 (8%) and new postoperative hearing dysfunction in 13 patients (11%). Seventeen patients (14%) with facial nerve dysfunction and 11 (9%) with auditory impairment showed an improvement. In 7 patients (6%), the function of the caudal CN worsened and became better in 1% of cases. Altogether, we saw additional postoperative CN deficits in 33 (27.3%), improvement in 40 (32.2%), and no change in 49 (40.5%) cases (Fig. 1).

### Recurrent petroclival meningiomas (n = 25)

Of 25 patients with recurrent PCM, 20 patients were diagnosed with a first recurrence, four with a second, and one patient with a third recurrence. The gender distribution male: female was 5:15, and the median age was 65.4 years. The median tumor size was 4.0 cm (range 2.8–6.7 cm). The median time to the first recurrence was 59.9 months (4.40–274.21) and from the first to the second recurrence 16.17 months (5.52–34 months). The time interval between the second and third recurrence was four months.

In the first recurrence, three of the meningothelial meningiomas transformed into an atypical meningioma WHO grade 2. One of the transitional tumors turned into an atypical meningioma. Upon the second recurrence, we diagnosed two meningothelial tumors, one atypical meningioma—which was already atypical in the first recurrence—and one atypical meningioma that had transformed into an anaplastic meningioma. Upon the third recurrence, we operated on an anaplastic meningioma WHO grade 3 that was previously an atypical. GTR was possible in three surgeries (12%), while in 22 we performed a subtotal removal with the goal of tumor mass reduction and postoperative radiotherapy in three cases.

#### (a) Preoperative symptoms in recurrent cases

Cerebellar dysfunction with ataxia, vertigo, dizziness (n = 17) and CN5 affections (n = 16) were the most diagnosed neurological deficits, followed by CN3 (n = 10) and CN8 nerve (n = 11) malfunctions. CN7 was affected in 6 patients, the CN6 in four, the CN4 in two, and CN9 in one patient. (Table 2).

**Table 2:** Preoperative neurologic deficits and at follow-up after recurrent PCM surgery CN: cranial nerve

Recurrent PCM n = 25 cases	Preoperative status	Follow-up status
Cranial nerve deficits [n (%)]		
CN 3	10 (40%)	11 (44%)
CN 4	2 (8%)	2 (8%)
CN 5	16 (64%)	16 (64%)
CN 6	4 (16%)	4 (16%)
CN 7	6 (24%)	8 (32%)
CN 8	11 (44%)	10 (40%)
CN 9	1 (4%)	2 (8%)
CN 10	–	–
Cerebellar dysfunction [n (%)]	17 (68%)	14 (56%)

#### (b) Postoperative outcome

In three patients, the ataxia improved postoperatively. One patient developed a new CN3 deficit. Two patients experienced worsening CN7 function and one patient experienced a new caudal CN deficit. Consequently, concerning only the CN function, 16% worsened, as shown in Table 2.

### Postoperative outcome according to the classification system using the retrosigmoid and the subtemporal/ pterional approach

According to our proposed classification system, the data of 123 tumor surgeries were available with regard to GTR, of which 104 were operated via the retrosigmoid and 19 via the subtemporal/pterional approach (Table 3). Small tumor size < 10 cm^3^ (p = 0.009) and soft tumor consistency (p < 0.001) were significant factors for complete tumor removal. In type I tumors, GTR was achieved in 78.7%, which was highly significant (p < 0.001) (Table 3). Regarding CN deficits, follow-up data of 112 tumor surgeries of 101 patients with an RSA (n = 96) and subtemporal/pterional approach (n = 16) were available. Patients with recurrent tumors were also included in the evaluation. In the univariate analysis, parameters associated with significantly favorable CN status were a soft tumor consistency (p = 0.038) and unilateral localization (p = 0.016). The smooth surface of the tumor with an arachnoid cleavage and the degree of vascularization did not play a significant role (Table 4). Patients with tumor type I had the best outcome with respect to CN deficits (p = 0.006) compared to all other types, preoperatively diagnosed CN deficits resolved in 35.6%, while a worsening of the symptoms occurred in 11.9% of cases (Table 4). The postoperatively observed CN deficits resolved in a high percentage over time. This was especially striking in the groups type II–IV (Supp Fig. 5).

**Table 3:** Univariate analysis demonstrating the association between tumor characteristics and GTR

123 Surgeries in 111 patients: 104 retrosigmoid, 19 subtemporal/pterional	GTR	p-value
Age [n = 111]		
≤ 60 years [n = 50]	70.0%	.242
> 60 years [n = 61]	59.0%	
Sex [n = 111]		
Male [n = 19]	52.6%	.299
Female [n = 92]	66.3%	
Two-stage surgery		
No [n = 111]	61.3%	.539
Yes [n = 12]	50.0%	
Cavernosus sinus infiltration [n = 123]		
No [n = 91]	71.4%	< .001
Yes [n = 32]	28.1%	
Tumor size [n = 105]		
≤ 10 cm^3^ [n = 54]	75.9%	.009
> 10 cm^3^ [n = 51]	51.0%	
Tumor consistency [n = 123]		
Soft [n = 73]	75.3%	< .001
Firm [n = 50]	38.0%	
Tumor surface [n = 123]		
Smooth [n = 83]	65.1%	.120
Cauliflower [n = 40]	50.0%	
Tumor location		
Unilateral [n = 92]	65.2%	.058
Bilateral [n = 31]	45.2%	
Vascularity		
Low [n = 88]	63.6%	.227
High [n = 35]	51.4%	
Proposed grading system [n = 123]		
Type I [n = 61]	78.7%	< .001
Type II [n = 12]	41.7%	
Type III [n = 31]	45.2%	
Type IV [n = 19]	36.8%

**Table 4:** Univariate analysis demonstrating the association between tumor characteristics and cranial nerve deficits

112 Surgeries in 101 patients: 96 retrosigmoid, 16 subtemporal/pterional	Cranial nerve deficits preoperatively vs. at follow-up					
	Preop. with CN deficits	p-value	CN status unchanged	CN status improved	CN status worsening	p-value at follow-up
Age						
≤ 60 years [n = 47]	63.8%	.287	46.8%	34%	19.1%	.464
> 60 years [n = 54]	74.1%		38.9%	31.5%	29.6%	
Gender						
Male [n = 16]	81.3%	.378	56.3%	25.0%	18.8%	.483
Female [n = 85]	67.1%		40.0%	34.1%	25.9%	
Two-stage surgery						
No [n = 100]	70%	1.0	43%	32%	25%	.293
Yes [n = 12]	75%		66.7%	16.7%	16.7%	
Cavernous sinus infiltration						
No [n = 89]	68.5%	.448	46.1%	31.5%	22.5%	.711
Yes [n = 23]	78.3%		43.5%	26.1%	30.4%	
Tumor size						
≤ 10 cm^3^ [n = 50]	66%	.828	44%	34%	22%	.769
> 10 cm^3^ [n = 46]	69.6%		39.1%	32.6%	28.3%	
WHO grade						
WHO grade 1 [n = 99]	69.7%	1.0	47.5%	29.3%	23.2%	.336
WHO grade 2 [n = 12]	75.0%		25%	41.7%	33.3%	
Tumor consistency						
Soft [n = 69]	68.1%	.528	50.7%	33.3%	15.9%	.038
Firm [n = 43]	74.4%		37.2%	25.6%	37.2%	
Tumor surface						
Smooth [n = 77]	70.1%	1.0	48.1%	31.2%	20.8%	.466
Cauliflower [n = 35]	71.4%		40%	28.6%	31.4%	
Tumor location						
Unilateral [n = 85]	70.6%	1.0	48.2%	34.1%	17.6%	.016
Bilateral [n = 27]	70.4%		37%	18.5%	44.4%	
Tumor vascularity						
Low [n = 81]	69.1%	.651	45.7%	28.4%	25.9%	.679
High [n = 31]	74.2%		45.2%	35.5%	19.4%	
Proposed grading system						
Type I [n = 59]	67.8%	.560	52.5%	35.6%	11.9%	.010
Type II [n = 10]	90.0%		40.0%	50.0%	10.0%	
Type III [n = 27]	70.4%		37.0%	18.5%	44.4%	
Type IV [n = 16]	68.8%		37.5%	18.8%	43.8%	
Type I [n = 59]	67.8%	.539	52.5%	35.6%	11.9%	.006
All other types [n = 53]	73.6%		37.7%	24.5%	37.7%	

### Adjuvant radiotherapy or radiosurgery

Postoperative radiotherapy or radiosurgery was applied to 12 patients. Eight patients were irradiated after their first surgery, of which five remained tumor-free, resp. without tumor progression. Four of the 12 patients received radiotherapy after tumor regrowth. One of them with a small recurrent tumor decided for radiosurgery in a cyberknife center. Adjuvant radiotherapy was mainly indicated in WHO grade 2 meningiomas with residual tumor and in grade 3 meningiomas.

### Radiographic evaluation of preoperative MRI and intraoperative tumor features

The evaluation of the T2 signal intensity of the tumor in comparison with the cortex to estimate tumor consistency on preoperative MR images demonstrated that tumors with a hyperintense signal in T2 were highly likely to have a soft texture with a sensitivity of 95.6% and a negative predictive value of 88.2%. Nevertheless, a subset of firm tumors had a hyperintense signal in the T2 criteria, as the specificity was 60%. The results of the analysis are shown in Table 5.

**Table 5:** Probability of soft tumor consistency based on hyperintensity signal in T2-weighted MR images

Statistic	Value (%)	95% CI (%)
Sensitivity	95.6	84.8–99.5
Specificity	60.0	38.6–78.8
Positive predictive value	81.3	72.6–87.4
Negative predictive value	88.2	65.1–96.8
Accuracy	82.86	72–90.8

### Complications after first surgery

A new hemiparesis occurred in three patients after primary surgery. Three patients suffered from dysphagia, two were treated with a tracheostomy. Six patients received external ventricular drainage due to hydrocephalus, of whom four had a permanent shunt implanted. One chronic subdural hematoma had to be evacuated. CSF fistula occurred in five patients, which could be resolved by insertion of a lumbar drainage or an operative revision. Altogether, our complication rate was 16.5%. Three patients died because of deep vein thrombosis with fulminant lung embolism, extra- and intracerebral infarctions, and septical multiorgan failure (mortality rate 2.3%) (Table 6).

**Table 6:** Rate of complications

	All surgeries (n = 158)	First surgery (n = 133)	Recurrent surgery (n = 25)
n (%)	27 (17.1%)	22 (16.5%)	5 (20%)
CSF leakage	8 (5%)	5	3
Hydrocephalus/shunt implantation	6 (3.8%)	4	2
Pulmonary embolism	3 (1.9%)	3	0
Hemiparesis	3 (1.9%)	3	0
Dysphagia	3 (1.9%)	3	0
Chronic subdural hematoma	1 (0.6%)	1	0
Postop. mortality	3 (1.9%)	3	0

### Complications after recurrent surgeries

After recurrent surgeries, three patients developed a CSFfistula, of which in two cases a ventricular shunt had to be implanted. The complication rate after recurrent surgeries was 20% with no severe complications.

## Discussion

The appropriate treatment of PCM is a matter of debate [2–14]. The risk of postoperative neurological deficits is high [2, 13, 19–28]. Besides surgery alternative treatments are considered like radiation or observation [29–32]. Since many of these lesions cause brainstem compression, surgical decompression is indicated, although the symptoms of the patients might only be mild. Over the last decades, the indication for surgery changed from aggressive GTR to subtotal or even “wait and scan” policies [13, 33]. Data about the natural course of the tumors are sparse. In 2003, van Havenbergh et al. concluded that the growth pattern of these tumors is unpredictable and that in relation to the infratentorial enlargement more neurological deficits occur [37]. Hunter et al. stated that the vast majority (88%) of these tumors grow [38]. According to both publications, active treatment seems to be necessary, at least as soon as tumor growth is observed.

Besides the advantages of complex skull base approaches, the workhorse for tumors of the cerebello-pontine angle is the retrosigmoid suboccipital approach, which can be combined with a tailored supratentorial approach in case of extension to the middle and anterior fossa [10, 12–14, 39]. These approaches were mainly used in our series.

The overall results of the whole study group of 133 primary PCMs are 27.3% new CN deficits and 59.4% GTR, which is in the range of major published series, in which different skull base approaches have been used [2–14, 22–31]. CN deficits are described in 22–67% and GTR was achieved between 28 and 63%. In a recent meta-analysis, Di Carlo et al. evaluated twelve studies with respect to CN deficits, dichotomized to the retrosigmoid and to petrosal approaches [40]. They found that CN7 palsy was more often observed in the RSA (16.6% vs 11.4%), whereas CN4 palsy occurred at a higher rate in the petrosal approach (7.6% vs 2.1%). This is consistent with our observation that CN7 and CN 8 have a relatively high risk for new deficits (8 and 11%) during PCM resection via the RSA, which is explained by their location within the surgical work path. Although the meta-analysis included only studies with a relatively low number of patients, the results are important for the preoperative risk stratification in regard to different approaches. Altogether, permanent CN deficits occurred in these studies in a low range and were superior to those data presented in larger series.

Our data include 25 patients with recurrent PCMs. Operating on these lesions has a restriction in itself. If the primary tumor was not completely resectable during the first surgery, one cannot expect to be more successful in the second session. If the tumor had a malignant grade, the risk for fast regrowth was high. Therefore, the indication for repeated surgery has to be critically evaluated and all were done on an individual basis. We saw an indication when significant regrowth of tumor remnants associated with brainstem compression was seen after the first surgery. The tumors were already irradiated and regrew. The tumors were WHO grade 2 and 3 meningiomas, had been already irradiated and no alternative treatment options were left. Most of the patients with regrowing PCMs suffer already from neurological deficits, as seen in our cohort. The risk for further deficits is therefore comparably low. As shown by Li in this special group, GTR was possible in eight out of 23 patients (34.8%) [41], and surgical morbidity occurred in 36.4%. GTR in our series was achieved in only three patients (12.5%) and the morbidity rate was 20%.

Our general strategy for any lesion in the brain is to stay primarily with a standard approach, tailored in size to each lesion. The experience of others [8, 13, 14, 23, 26, 28, 40] and our own showed us that four main factors influenced the resectability of the PCMs and that these factors also influenced the outcome of the patients (1) the localization with the dural adhesion zone, (2) the consistency of the tumor, (3) the surface of the tumor, and (4) the arachnoid cleavage plane and the lack of brainstem edema. According to our results, the leading intraoperative factor was tumor consistency. Little et al. (2005) described already tumor consistency as an important factor for resectability [28] by pointing out that fibrous tumors have an increased risk because of the need for sharp dissection and tumor manipulation during debulking. In this meningioma type, cranial nerve deficits occurred in his series in 41%, which was much higher than in all others. Kawase raised the question in his comment about the possibility to diagnose relevant tumor-specific properties prior to surgery [28].

Influenced by these data, we propose a classification system for the extent of resection and CN outcome, by combining the aspects of anatomical localization and specific tumor features, as mentioned above. We focused our classification-based analysis primarily on standard approaches such as the RSA and the subtemporal/pterional craniotomy. By stratifying our results to the proposed classification system, it became obvious that significantly better results of CN preservation could be achieved in type I and type II tumor patients, in which new cranial nerve deficits occurred in only 11.9% resp. 10.0% which was significantly better in comparison to the groups III and IV with 44.4 and 43.8% (p = 0.010). Notably, GTR was achieved in 78.7% of type 1 tumors, which was significantly higher than in the types II-IV (p < 0.001). And again, the most important factor for a successful and uneventful resection was the consistency of the tumor with an arachnoid cleavage plane to the brainstem. The vascularity did not play a significant role in the preservation of CN functions, as anticipated. Therefore, we did not include vascularity as a further factor in our final classification system. Preoperative embolization of highly vascularized lesions was also not performed by the neuroradiologists in our series due to possible embolization-associated complications [42].

The ideal goal for the further development of the intraoperative classification system, as used, is the determination of a patient’s risk of undergoing surgery prior to the procedure. Therefore, tumor-specific features should be definable on preoperative MRI. In recent years MRI parameters have been identified to group meningiomas that are easier or less resectable. Pirayesh et al. reported about an excellent correlation between tumor resectability and preoperative presence or absence of an arachnoid cleavage plane and an irregular tumor margin towards the brainstem [20]. Nicosia et al. pointed out that a high tumor intensity on a T2-weighted image correlates with soft tumor consistency and vascularity, whereas brainstem edema is significantly related to surgical morbidity because of invasive growth [20, 21]. From these findings, we assessed our data with respect to preoperative MRI scans. Our special focus was on the consistency of the tumors. Preoperative MR images of 70 patients were available for evaluation. Consistent with prior reports, tumors with a hyperintense signal in T2 were highly likely to have a soft texture with a sensitivity of 95.6% and a negative predictive value of 88.2%. Our results corroborated the previous findings and corresponded well with the proposed features. Consequently, to build on these results, we have initialized a prospective study with systematic data collection on a standard protocol that allows us to use the radiological information for better planning and risk stratification in PCMs.

Over time, the knowledge about the PCM-specific properties changed our surgical understanding. We tried to achieve GTR in type I and, if possible, in type II PCMs and went for mass reduction in type III and type IV tumors as much as possible without taking risks for the quality of life of the patients, a strategy that has been also advocated by Seifert in his landmark paper [13]. Especially in type IV tumors, which are more clival meningiomas than PCMs the experience taught us that these tumors are of high risk for neurological deficits and can barely be removed via standard approaches.

Major complications in our series were new permanent hemipareses in three cases, which were in two cases associated with dysphagia that urged us to tracheotomize the patients. Severe complications occurred predominantly in patients with firm tumors or aggressive meningiomas grade 2 and 3. With a complication rate of 16.5% in primary and 20% for recurrent PCM surgery, we are in the middle of the overall common complication or morbidity rate of 11 – 45%, described in the literature [1, 13, 25, 27, 30, 33, 43, 44]. The mortality rate of PCM surgery is low and ranges between 0 and 10% [1, 13, 25, 27, 30, 33, 43, 44]. Our mortality rate of the whole group was 1.9% and occurred only in primary PCMs.

### Limitations

The presented study is a retrospective analysis. Therefore, not all data were available and follow-ups were missing. Regarding the outcome analysis, we present our experience with the main focus on the retrosigmoid and the subtemporal/pterional approach and specific tumor features, implemented in an intraoperative classification system. The logical consequence of this approach is a preoperative reliable risk stratification that allows the determination of specific tumor characteristics by MRI prior to surgery. A comparison between standard approaches and skull base approaches with a special focus on tumor-specific properties is pending to select the appropriate surgical approach on an individual basis.

## Conclusion

Standard approaches such as the retrosigmoid and the subtemporal/pterional approaches are suitable for most of the PCM surgeries. The advantages are the familiarity and simplicity of the procedure. If necessary, a combined approach can be used for tumor parts in the middle and anterior fossa. Because of the anatomical facts, the CN7 and CN8 are at higher risk for postoperative damage using the RSA compared to other skull base approaches. The most important features for a successful removal of the tumors are unilateral localization and a soft tumor consistency with an arachnoid cleavage plain. Firm tumor consistency and bilateral adhesion zones are less suitable for the RSA and demand obviously more complex skull base approaches. An MRI-related classification of specific tumor features prior to surgery might help to select the individual appropriate approach.

## Supplementary Information

Below is the link to the electronic supplementary material.

Supplementary file1 (DOCX 3450 KB)
