# Supplementary material for: Surgical results of 158 petroclival meningiomas with special focus on standard craniotomies
Source: J Neurooncol. 2022 Sep 14;160(1):55–65. doi: 10.1007/s11060-022-04105-5 (PMC9622548; doi:10.1007/s11060-022-04105-5)
Supplement: Supplementary file 1 — Supplementary file1 (DOCX 3450 KB) [file 11060_2022_4105_MOESM1_ESM.docx]

**Supplementary materials:**

**
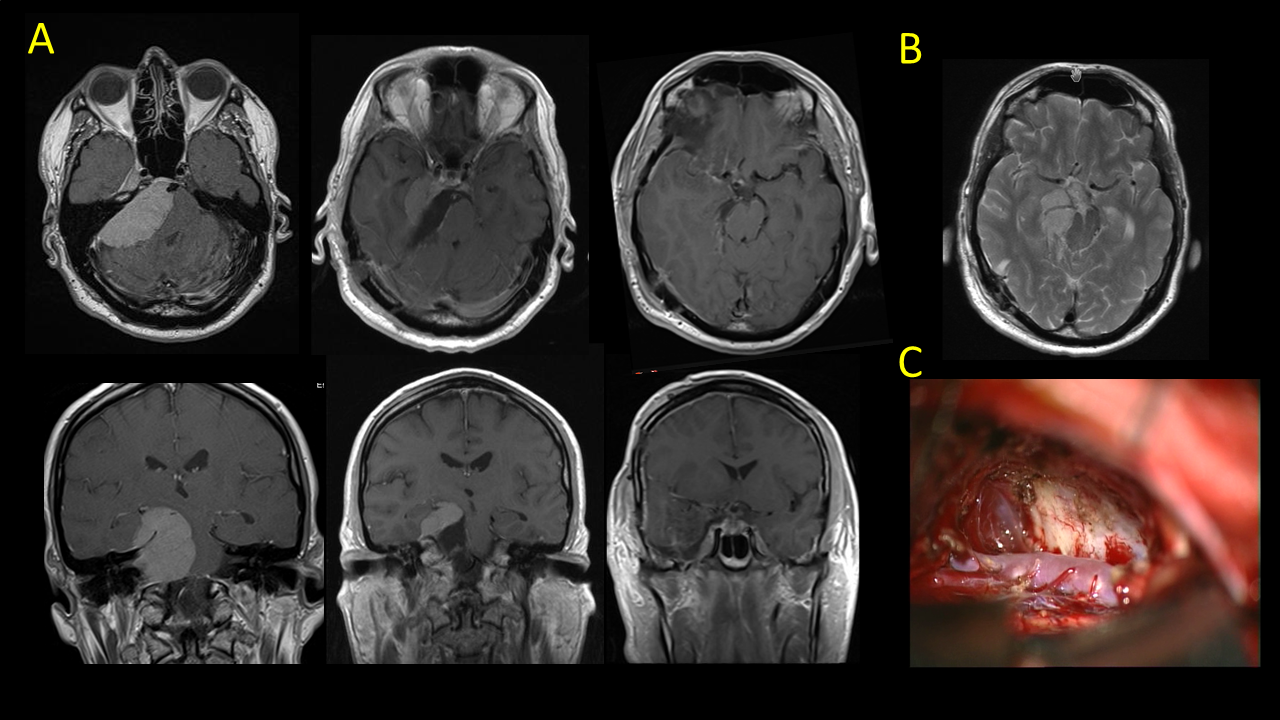
Supp. Figure 1: A)** Representative gadolinium-enhanced T1-weighted MRI of a 67-year-old patient who underwent a two-stage surgery to remove a PCM that extended to the middle fossa. **B)** The T2-weighted MRI reveals a tumor hyperintensity as a sign of a soft tumor consistency, which was confirmed intraoperatively. **C)** The intraoperative screenshot shows the basilar artery with its *rami ad pontem* after tumor removal. **Classification type I.**

**
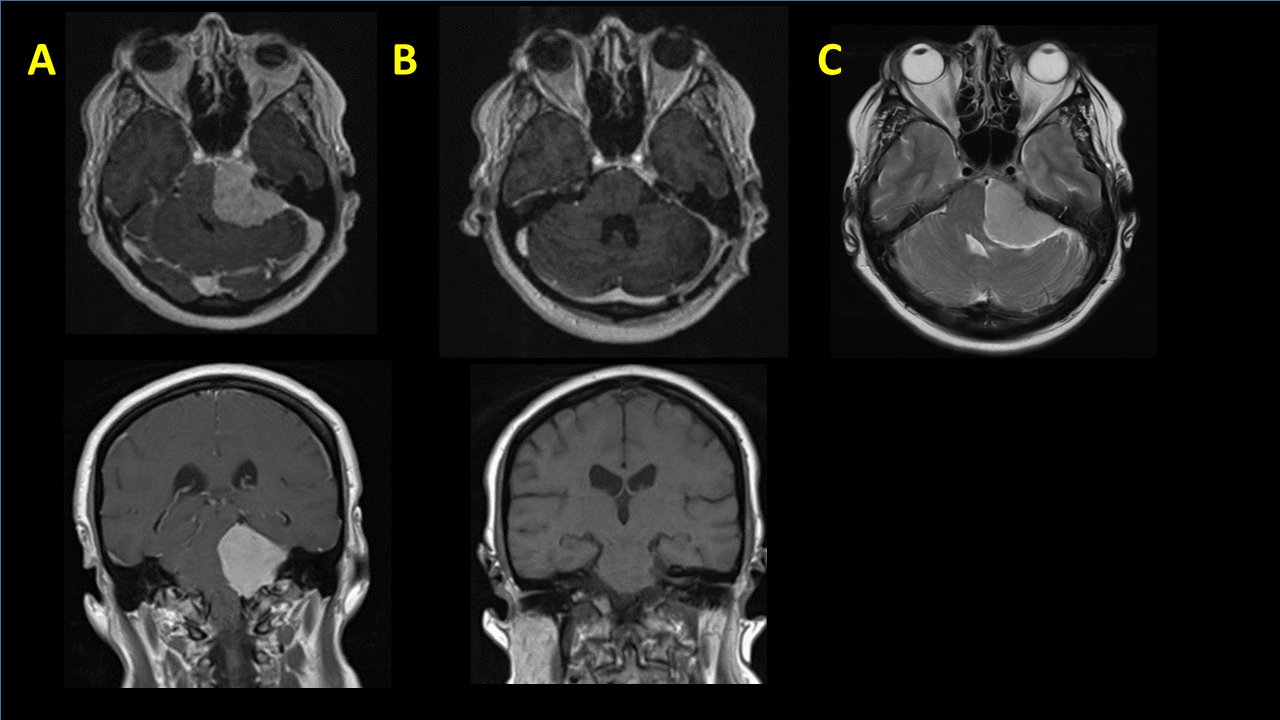
Supp. Figure 2: A)** Pre- and **B)** postoperative gadolinium-enhanced T1-weighted MR-imaging of a representative PCM type II in a 51-year-old woman. **C)** In preoperative T2-weighted MRI, the tumor shows a higher intensity than the cortex. Intraoperatively, the tumor was firm, but could easily be cut into pieces and removed. There was a nice arachnoid cleavage between the tumor, the brain stem, and the vessels. **Classification type II.**

**
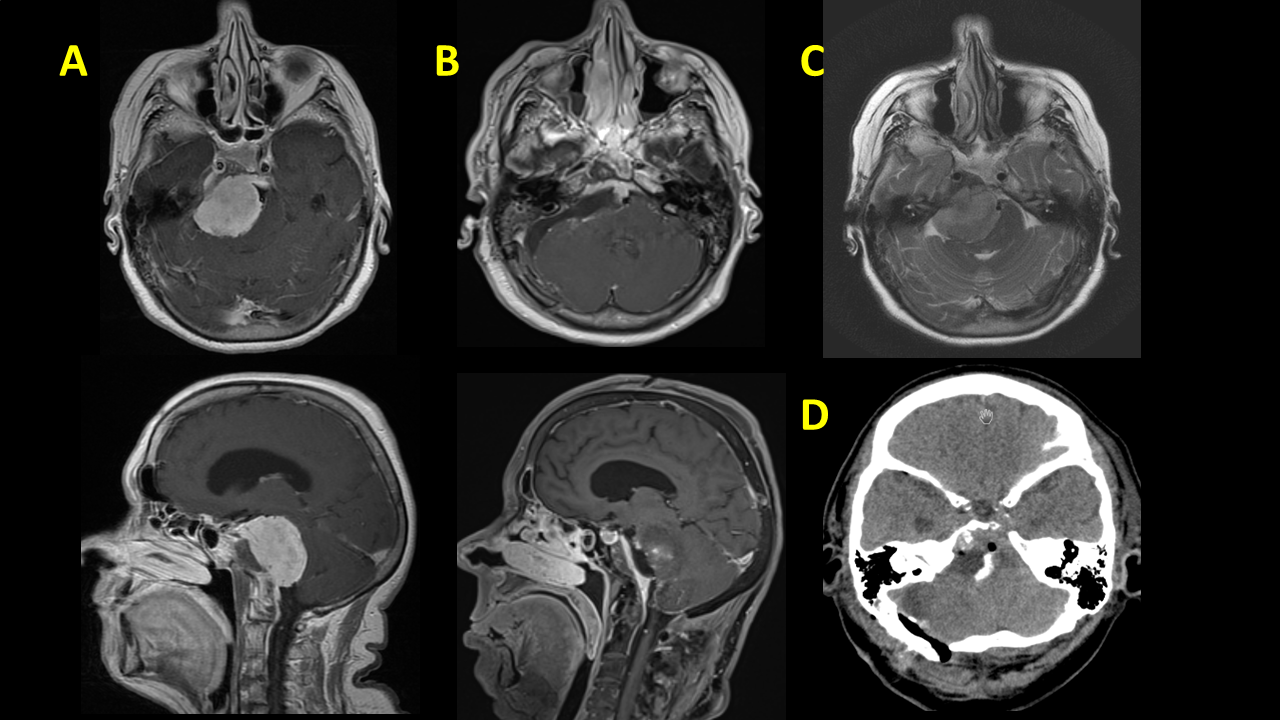
**

**Supp. Figure 3:** **A)** Representative gadolinium-enhanced T1-weighted pre- and **B)** postoperative MR imaging of a 68-year-old female patient with PCM. **C)** The tumor shows low T2-signal and no clear arachnoid cleavage between tumor and brain stem, suggesting a firm tumor consistency. **D)** The tumor was partially calcified and a tumor remnant was left behind in the midline, which could not be dissected from the vessels (CT postoperatively). The patient had a severe course with hemiparesis and tracheotomy. **Classification type III**

**
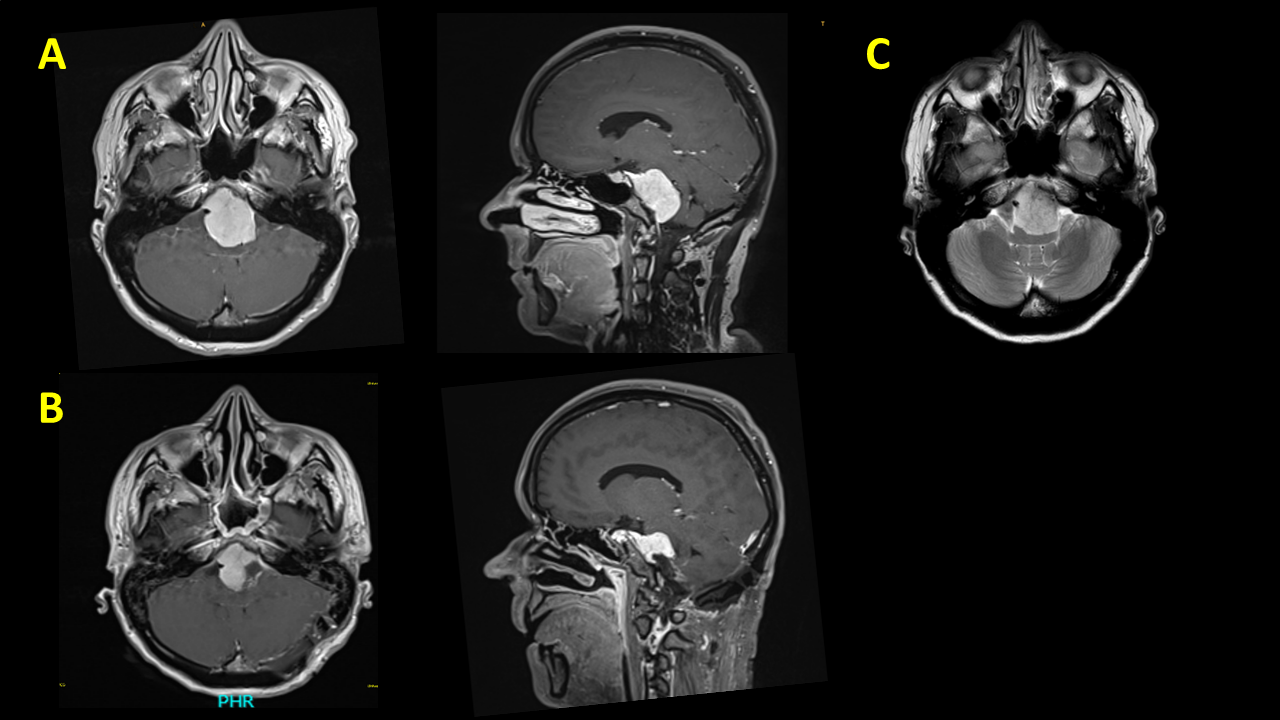
Supp. Figure 4:** **A)** Representative gadolinium-enhanced T1-weighted pre- and **B)** postoperative MRI of a 44-year-old woman with one-stage surgery via the left-sided retrosigmoid suboccipital approach. **C)** The preoperative T2-weighted MRI shows mostly high signal intensity of the tumor, suggesting that the tumor might be – at least partially - of soft consistency. The brainstem was maximally compressed. Intraoperatively, the tumor was of firm consistency. Only partial removal was possible via the RSA. Postoperatively, the patient required a transient tracheostomy. **Classification type IV**


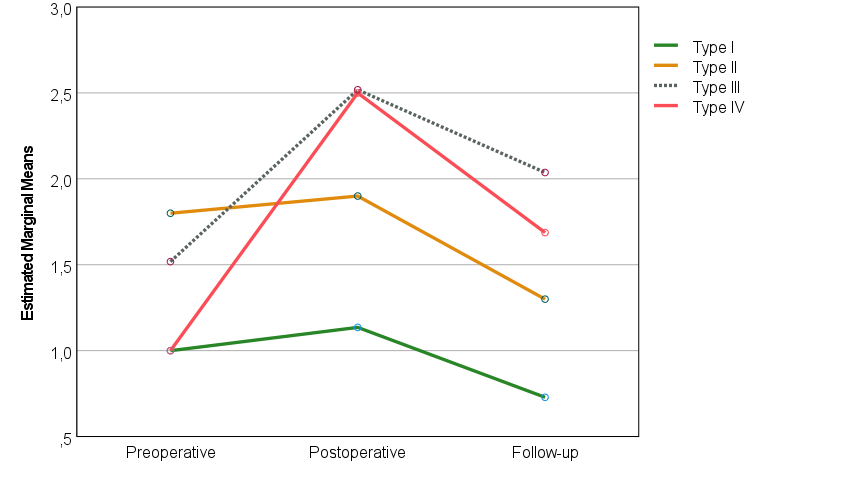


**Supp. Figure 5**: Estimated Marginal Means from the general linear model with measurement repetition. A large subset of patients with PCM of all types experienced cranial nerve deficits immediately postoperatively compared to preoperatively. However, this decreased to a substantially lower level at the time of the last clinical follow-up, suggesting that any neurological worsening following surgery is temporary in the majority of cases
